# Developmental trajectories of eating disorder symptoms: A longitudinal study from early adolescence to young adulthood

**DOI:** 10.1186/s40337-022-00603-z

**Published:** 2022-06-20

**Authors:** Édith Breton, Rachel Dufour, Sylvana M. Côté, Lise Dubois, Frank Vitaro, Michel Boivin, Richard E. Tremblay, Linda Booij

**Affiliations:** 1 Sainte-Justine Hospital Research Centre, Montreal, Canada; 2 Department of Psychiatry and Addictology, University of Montreal, Montreal, Canada; 3 Department of Psychology, Concordia University, 7141 Sherbrooke Street West, Montreal, QC H4B 1R6 Canada; 4 School of Public Health, University of Montreal, Montreal, Canada; 5 School of Epidemiology and Public Health, University of Ottawa, Ottawa, Canada; 6 School of Psychoeducation, University of Montreal, Montreal, Canada; 7 Department of Psychology, University Laval, Quebec, Canada; 8 Department of Psychology and Pediatrics, University of Montreal, Montreal, Canada; 9 Department of Psychiatry, McGill University, Montreal, Canada

**Keywords:** Eating disorder symptoms, Sex-specificity, Developmental trajectories, Adolescence, Mental health

## Abstract

**Background:**

Adolescence is a critical period for the development of eating disorders, but data is lacking on the heterogeneity of their evolution during that time-period. Group-based trajectories can be used to understand how eating disorders emerge and evolve over time. The aim of this study was to identify groups of individuals with distinct levels of eating disorder symptoms between 12 and 20 years and the onset of different types of symptoms. We also studied sex differences in the evolution and course of eating disorder symptoms from early adolescence to adulthood.

**Methods:**

Using archival data from the QLSCD cohort, trajectories of eating disorder symptomatology were estimated from ages 12 to 20 years using semiparametric models. These trajectories included overall eating disorder symptomatology as measured by the SCOFF (Sick, Control, One Stone, Fat, Food), sex, and symptom-specific trajectories.

**Results:**

Two groups of adolescents following distinct trajectories of eating disorder symptoms were identified. The first trajectory group included 30.9% of youth with sharply rising levels between 12 and 15 years, followed by high levels of symptoms between 15 and 20 years. The second trajectory group included 69.1% of youth with low and stable levels of symptoms between 12 and 20 years. Sex-specific models indicated that the proportion of girls in the high trajectory group was 1.3 times higher than the proportion of boys (42.8% girls vs. 32.3% boys). Trajectories of SCOFF items were similar for loss-of-control eating, feeling overweight, and attributing importance to food. The weight loss item had a different developmental pattern, increasing between 12 and 15 years and then decreasing between 17 and 20 years.

**Conclusions:**

The largest increase in eating disorder symptoms in adolescence is between the ages of 12 and 15 . Yet, most prevention programs start after 15 years of age. Our findings suggest that, unlike common practices, eating disorder prevention programs should aim to start before puberty.

**Supplementary Information:**

The online version contains supplementary material available at 10.1186/s40337-022-00603-z.

## Plain English summary

Eating disorders, typically involving preoccupation with eating, excessive exercise, and body image issues, are particularly common in adolescence. However, their evolution over time remains unclear as certain signs and symptoms may appear sooner than others. The current study studied the development of eating disorder symptoms in a community cohort followed from birth to adulthood. This study describes trajectories of eating disorder symptoms and risk from age 12 to 20 in both boys and girls. Results showed that the largest increase in eating disorder symptoms occurs between 12 and 15 years of age, both in overall symptomatology as well as in specific symptoms such as loss-of-control when eating, feeling overweight, and attributing importance to food. Additionally, more girls than boys appeared at risk for eating disorders, although the patterns represented by the trajectories were similar in both sexes. Our results highlight the importance of starting early prevention programs before the beginning of adolescence, when symptoms usually start to manifest.

**Supplementary Information:**

The online version contains supplementary material available at 10.1186/s40337-022-00603-z.

## Introduction

Adolescence is a critical time period for the development of eating disorders (EDs). EDs are present in 1–3% of adolescents, while around 30% of adolescent girls and 15% of adolescent boys present with disordered eating at a subclinical level [1–3]. Moreover, EDs are debilitating psychiatric disorders associated with several negative outcomes, and their typical symptoms include preoccupation with eating, compensatory behaviors, and body image disturbances [1, 2]. Not only can EDs lead to decreased quality of life, problems at work and school, and social isolation, but they are often associated with several psychiatric comorbidities (e.g., depression, anxiety) as well as physical health problems and even death [4]. Consequently, there is a pressing need to better understand how ED symptoms develop and how they lead to full-blown EDs.

### Sex differences in eating disorders

Research has shown differences between sexes when it comes to ED symptom presentations and risk factors [5–10]. Of note, biological sex and psychosocial gender have often been used interchangeably in the literature and although the two constructs are closely related, they should be distinguished. Therefore, we would like to clarify that the terms “men”, “women”, “boys”, and “girls” will be used to refer to the biological construct that is sex throughout the present manuscript. On the one hand, men are more likely to have Binge Eating Disorder (BED) than other EDs, to engage in more exercising and fasting, to have less body image distortions, and to have higher body mass index (BMI) and obesity rates than women [5–7]. On the other hand, in community samples, women are approximately four times more likely to have Anorexia Nervosa (AN) and Bulimia Nervosa (BN) than men, with this number increasing to ten times more likely in clinical settings [11]. In addition to symptom presentation, at least a few ED risk factors differ between sexes. For example, a longitudinal study found that childhood body dissatisfaction predicted weight and shape concerns in girls, while it only predicted weight and shape concerns in boys when interacting with BMI [8]. However, findings are mixed when it comes to other ED risk factors during adolescence; all sexes are affected by weight/appearance anxiety, use of tobacco, and poor communication with parents, among other factors [9, 10]. Given that the clinical presentation of EDs is often different in boys and men, sex-specific developmental patterns before ED onset should be clarified.

### Development of eating disorder symptoms

The evolution and development of ED symptoms need to be further studied, as it is unclear how early certain symptoms develop and cluster over time. Eating behaviors in childhood and preadolescence might represent early risk factors for later development of EDs in adulthood [12–16]. Additionally, some studies have shown that bingeing and purging-type symptoms (such as those present in BED and BN) appear later than restrictive symptoms (such as those present in AN) [17, 18]. Group-based developmental trajectories analyses represent a powerful way to better understand the development of EDs. Developmental trajectories have been used to study the evolution of BMI and weight status in youth [19, 20]. It is also possible to investigate the association between such trajectories and the onset of ED symptoms [20]. Studies on trajectories of ED symptoms suggest that symptomatology increases during adolescence, which is consistent with the typical onset of the first symptoms of AN and BN [7, 21]. Another study focusing on ED risk from 12 to 19 years identified three trajectory groups, interpreted as low risk, early onset, and late-onset ED risk trajectories [22].

Reviews have shown that prevention for EDs (e.g., the *Body Project* intervention), targeting the general population, tends to focus on body image as opposed to other components of EDs, and tends to start in high school. As these interventions typically result in modest reductions of symptoms, clarifying aspects of ED development would have important implications on preventive interventions [23, 24]. Still, there is a lack of research on developmental trajectories of ED symptoms in large community samples including both boys and girls, as previous studies mainly focused on clinical samples or exclusively relied on girls and women participants [12–18, 20]. Additionally, it is still unclear when and how different ED symptoms appear during adolescence in the general population.

### Aims of the study

The overarching aim of this study was to identify trajectory groups of individuals with distinct levels of ED symptoms during adolescence, in a large longitudinal community sample representative of the Quebec (Canada) population. The first aim was to describe heterogeneity (i.e., presence of diverse trajectory subgroups diverging in ED symptom evolution) in overall ED symptomatology in the Quebec Longitudinal Study of Child Development (QLSCD) cohort from ages 12 to 20. Second, the trajectories were modeled separately for each sex in order to assess potential sex-specificity in developmental patterns between boys and girls. Finally, trajectories for specific ED symptoms from early adolescence to early adulthood were analyzed to assess heterogeneity. Results might help to obtain a better understanding of the development of ED symptoms over time in adolescence.

## Methods

### Participants

This study used archival data from a community-based longitudinal cohort, the Quebec Longitudinal Study of Child Development (QLSCD). Details on the QLSCD design and data collections have been published elsewhere [25, 26]. Briefly, 2123 participants were recruited randomly at the age of 5 months through the Quebec Master Birth registry, in 1997–1998 [25, 26]. Follow-ups took place every one to two years and involved collecting data about the child’s health, psychosocial development, and environment. The time points of interest in the present study were at ages 12, 15, 17, and 20, where a questionnaire that evaluated mental health [27], including ED symptoms, was completed by the participants. See Orri et al. [26] for more details about the characteristics of the cohort.

This study was approved by the Health Research Ethics Committees of the Quebec Statistics Institute and the Sainte-Justine Hospital Research Center Ethic Committee. The participants themselves or their primary caregivers signed a written informed consent form before each data collection.

### Eating disorder risk

The Sick, Control, One stone (i.e., weight loss), Fat, Food (SCOFF) questionnaire was administered to assess ED symptoms [28, 29]. The present study is the first to report analyses based on the SCOFF in adolescents from the QLSCD cohort. The SCOFF includes the five following items: (1) Do you make yourself sick because you feel uncomfortably full? (i.e., purging) (2) Do you worry that you have lost control over how much you eat? (i.e., loss-of-control eating) (3) Have you recently lost more than 6 kg in a 3-month period? (i.e., weight loss) (4) Do you believe yourself to be fat when others say you are too thin? (i.e., feeling overweight) (5) Would you say that food dominates your life? (i.e., attributing importance to food) [28, 29]. The SCOFF total score reflects the number of ED symptoms and ranges from 0 to 5 [28, 29]. Questions are typically answered by “yes (1)” or “no (0)”, with a score of two or more being considered as a risk for EDs [28, 29]. This cut-off of 2 has been established and considered clinically relevant based on previous studies about the psychometric properties of the SCOFF [28, 29]. Throughout the manuscript, the terms “high levels” and “low levels” of ED symptoms refer to this cut-off of 2/5 (i.e., individuals with high levels of symptoms are those with a score of two or more at the SCOFF).

While the original version of the SCOFF was administered at age 12, a slightly modified version was used at ages 15, 17, and 20 to allow for a more thorough understanding of the severity of ED symptoms. At these three time points, participants’ responses were collected based on a Likert scale (i.e., never (0), sometimes (1) or often (2)). One exception to this was for the 3^rd^ question (i.e., have you recently lost more than 6 kg in a 3-month period?) at 20 years, where the typical binary scale was used. In order to study the evolution of the ED symptom across each measurement time, and to be more comparable with previous studies using the SCOFF, the Likert scale used at ages 15, 17 and 20 was re-coded as follows: never = no (0); sometimes or often = yes (1). This re-coding allowed the calculation of the ED symptom levels variable, classifying adolescents as having “high level” (1) or “low level” (0) of ED symptoms, with the cut-off set at 2/5 [28, 29]. These binary ED symptom levels variables from 12 to 20 years were used to model the trajectories.

### Missing values

At age 12, 1336 participants (52.2% girls) completed the SCOFF. At 15 years, this number was 1443 (52.2% girls), then 1269 (54.1% girls), and 1243 (57.8% girls) at ages 17 and 20, respectively. Data were not completely missing at random as boys were more likely to be missing a value than girls. The semiparametric analyses presented below are designed to handle missing data when modeling the trajectories using the maximum likelihood method of estimation [30]. Thus, youths were included in the analyses if their ED symptoms were assessed at least once between 12 and 20 years. In sum, 872 participants provided data at four time points, 417 at three time point, 193 at two time point, and 166 at one time point.

### Statistical analyses

A semiparametric procedure in the Proc Traj program of SAS (version 9.4), also known as latent class growth analysis, was used to model the different groups of ED symptom trajectories within the QLSCD cohort. This procedure was used without any a priori hypotheses. Proc Traj provides an estimate of the proportions of individuals in the study population following each trajectory group, as well as the probabilities that each individual has to belong to the different trajectory groups [30]. Participants are then assigned to the trajectory group to which they have the highest probability of belonging to. The Bayesian Information Criterion (BIC) guided the choices when optimizing the models, with a BIC closer to 0 being an indicator of a better model fit [30]. Proc Traj default start value was used to estimate the trajectories.

In order to optimize the replicability of the results, the GRoLTS checklist for reporting latent class trajectory study was used [31]. Time was coded in the models as years (12, 15, 17 and 20); however, the use of archival data did not allow us to control for possible time-unstructuredness. Graphs for each model estimated during the final model’s selection process, as well as syntax files can be made available upon request.

First, ED symptomatology trajectories for the entire cohort were modeled using the SCOFF score (i.e., high level vs. low level of symptoms). In addition, an alternative approach was used, and the trajectory model was run without the weight loss item of the SCOFF. The removal of the weight loss item led to a stricter criterion for having “high level of symptoms”, as the threshold of two was kept (i.e., the scores ranged from 0 to 4 instead of 0 to 5). This decision was made since the weight loss item refers to significant weight loss in a short period of time, a symptom characteristic of AN which has a lower prevalence than other EDs [32, 33]. Consequently, the weight loss item may not be the most representative or frequent ED symptom in the QLSCD cohort. Second, analyses were performed separately for each sex. Third, trajectories were modeled for each individual item of the SCOFF.

In order to identify the ideal number of trajectory groups, models with different numbers of groups were tested (i.e., 1, 2, 3 and/or 4 groups). Then, different trajectory shapes (i.e., stable, linear, and quadratic) were tested to identify the models that would best fit the data. With binary outcomes like those used in the current study, the Proc Traj procedure estimates the probability of having the behavior at each measurement time, in this case the probability of having high levels of ED symptoms.

## Results

### Demographics

Table 1 shows the demographic distribution (sex, ethnicity), and the proportions of ED symptoms at each time point. Across time points, the sample consisted of approximately 54% girls, and was primarily of Canadian/European origin (81.9%).

**Table 1:** Participant characteristics and ED symptoms (SCOFF items) from early adolescence to early adulthood

Variable *n* (%)	12 years *n* = 1336	15 years *n* = 1443	17 years *n* = 1269	20 years *n* = 1243
Sex, *n* boys/girls (%)	638/698 (47.8/52.3)	690/753 (47.8/52.3)	582/687 (45.9/54.1)	525/718 (42.2/57.8)
Ethnic origin, *n* (%)				
Canadian/European	1104 (82.6)	1186 (82.1)	1036 (81.6)	1010 (81.3)
American–Indian	41 (3.1)	40 (2.8)	43 (3.4)	35 (2.8)
African, Haitian	22 (1.7)	24 (1.7)	24 (1.9)	25 (2.0)
Other	162 (12.1)	183 (12.7)	161 (12.7)	167 (13.4)
Unknown	7 (0.5)	10 (0.7)	5 (0.4)	6 (0.5)
ED risk *n* (%)	299 (22.4%)	489 (33.9%)	447 (35.2%)	124 (10%)
Sick *n* (%)	204 (15.2%)	54 (3.7%)	67 (5.3%)	64 (5.1%)
Control *n* (%)	240 (17.8%)	561 (38.9%)	486 (38.4%)	475 (38.2%)
One stone *n* (%)	186 (13.9%)	202 (15.9%)	227 (15.7%)	125 (10.1%)
Fat *n* (%)	167 (12.4%)	314 (21.8%)	244 (19.2%)	207 (16.7%)
Food *n* (%)	335 (24.9%)	649 (45%)	632 (49.9%)	500 (40.2%)

Sick, Control, One Stone, Fat, and Food refer to the five items of the SCOFF questionnaire. ED risk indicates the number of people (%) that has at least 2 items on the SCOFF questionnaire are scored positive. The numbers listed for the Sick, Control, One stone, Fat or Food item indicates the number of people (%) scoring positive on the item of interest

### Trajectories of overall symptom levels

**Fig. 1:**
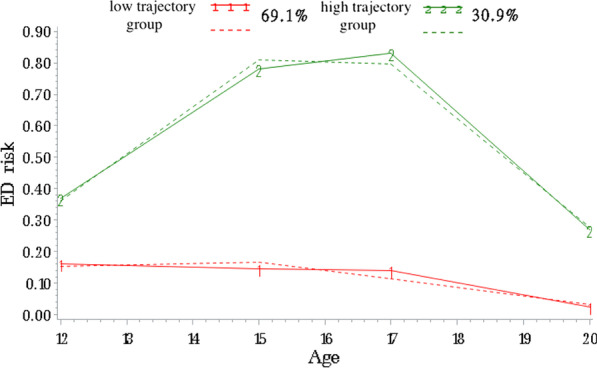
ED risk trajectories from 12 to 20 years of age, including both sexes. The dashed lines represent estimated values, whereas the solid lines represent observed values

Table 2 shows the process of identifying the model with the best fit for ED symptoms in the entire sample and by sex. The most optimal model was a model with two trajectory groups defined by a quadratic equation (*P* < 0.01). There was a high symptom levels trajectory group (30.9% of the cohort) and a low symptom levels trajectory group (69.1% of the cohort). Mean posterior probabilities were between 0.83 and 0.89, indicating a good model fit, based on the guidelines of Nagin et al. [27]. The high-level trajectory group already had higher ED symptom levels at age 12. The low-level trajectory group remained low throughout the follow-up period, and it showed a slight decrease over time. Figure 1 shows the model with the best fit for the evolution of ED symptom levels in the cohort. Additional file 2: Figure S1 has more details on the numerical characteristics of the final models.

**Table 2:** Model fit for eating disorder symptom levels trajectories

Model	# of trajectory groups	Order (0 = constant, 1 = linear, 2 = quadratic)	BIC
(A) *Full sample*			
Step 1: identify the ideal number of trajectory groups			
1	3	2 2 2	− 2748.6
2	4	2 2 2 2	− 2762.04
**3**	**2**	**2 2**	− **2734.8**
4	1	2	− 2879
Step 2: identify the ideal shape for the trajectories			
**3**	**2**	**2 2**	− **2734.8**
5	2	1 2	− 2735.9
(B) *Only girls*			
Step 1: identify the ideal number of trajectory groups			
1	3	2, 2, 2	− 1595.21
**2**	**2**	**2**, **2**	− **1579.29**
3	1	2	− 1661.45
Step 2: identify the ideal shape for the trajectories			
**2**	**2**	**2**, **2**	− **1579.29**
4	2	1, 2	− 1582.03
5	2	2, 1	− 1634.24
(C) *Only boys*			
Step 1: identify the ideal number of trajectory groups			
1	3	2, 2, 2	− 1109.16
**2**	**2**	**2**, **2**	− **1093.35**
3	1	2	− 1118.05
Step 2: identify the ideal shape for the trajectories			
2	2	2, 2	− 1096.62
**4**	**2**	**1**, **2**	− **1093.35**
5	2	0, 2	− 1103.28
6	2	1, 1	− 1115.36

Selected models are displayed in bold

*BIC* Bayesian Information Criterion

### Trajectories of overall symptom levels without the weight loss item

With this alternative approach, the model with the best fit had two trajectory group, one was a constant (low-level) and the other was quadratic (high-level) (*P* < 0.0001). This model had a good fit, with mean posterior probabilities between 0.84 and 0.88. Excluding the weight loss item led to similar proportions of individuals in each group of trajectories for overall ED symptomatology compared to the model including the weight loss item. In the model excluding the weight loss item (Additional file 1), the high-level trajectory group was more stable from ages 17 to 20 than in the models including this item (Fig. 1). This was also verified by a McNemar test (SPSS), which revealed a significant difference in ED symptom levels between 17 and 20 years when the weight loss item (*P* < 0.001) was included but not when it was excluded (*P* > 0.05).

### ED symptoms in boys and girls

**Fig. 2:**
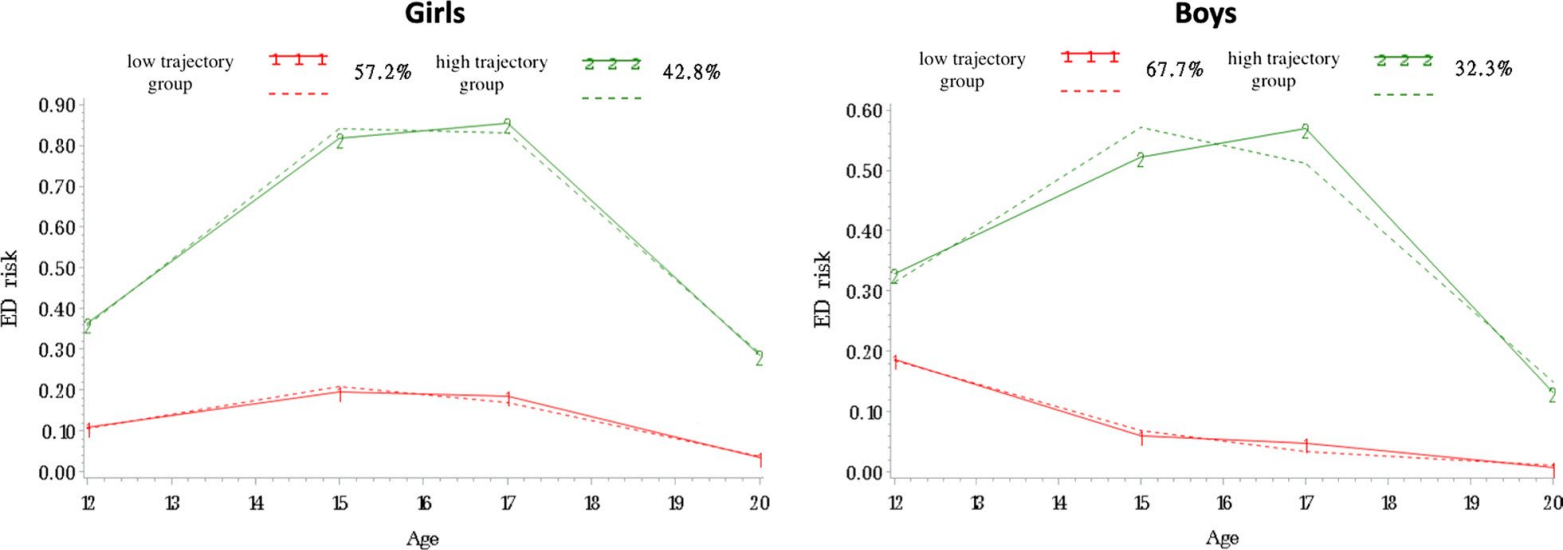
ED risk trajectories from 12 to 20 years of age for girls (**A**) and boys (**B**). The dashed lines represent estimated values, whereas the solid lines represent observed values

As proportion of girls in the high-level trajectory was higher than for boys (chi-square *P* < 0.0001), ED symptoms trajectories were tested for boys and girls separately. Figure 2 shows the selected models for ED symptoms for girls (Fig. 2A) and boys (Fig. 2B). In these two models, two trajectory groups were identified (see Table 2). For girls, the model with the best fit had two significant quadratic trajectory groups (*P* < 0.0001). For boys, the model with the best fit had one linear (low-level, *P* = 0.02) and one quadratic (high-level, *P* < 0.0001) trajectory groups. The proportion of individuals in each trajectory group was as follows: for girls, 57.2% in the low-level group and 42.8% in the high-level group; for boys, 67.7% in the low-level group and 32.2% in the high-level group. This means that the proportion of girls with high levels of ED symptoms was 1.3 times higher than the proportion of boys with high levels of ED symptoms. Within the high-level trajectory groups, the risk of ED did not peak as high for boys as it did for girls. These models have good fits, with mean posterior probabilities between 0.79 and 0.86.

### Trajectories of specific ED symptoms

**Fig. 3:**
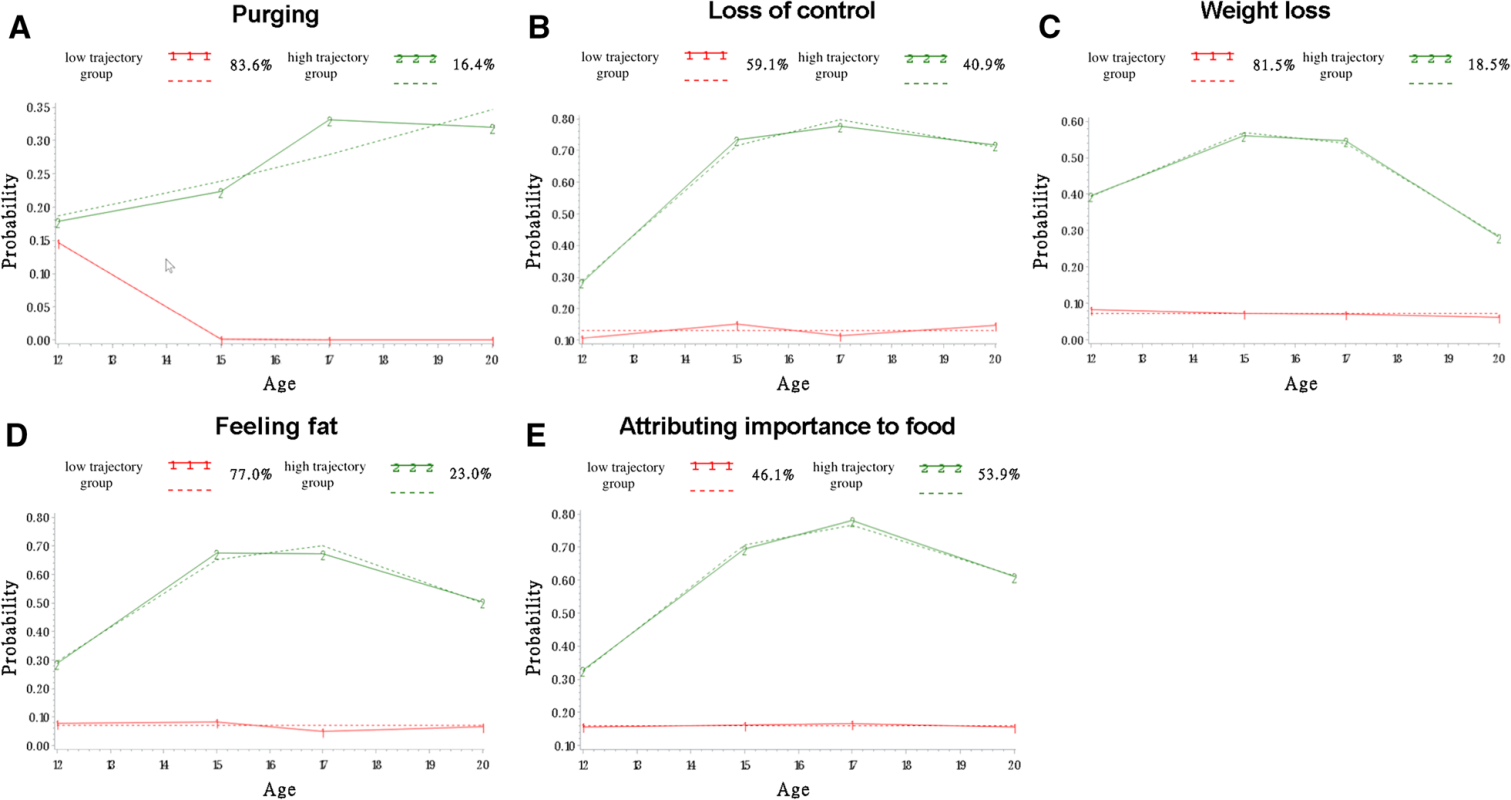
Trajectories for each symptom of eating disorders from 12 to 20 years of age for both sexes. **A** Purging, **B** loss-of-control eating, **C** weight loss, **D** feeling overweight, and **E** attributing importance to food. The dashed lines represent estimated values, whereas the solid lines represent observed values

Figure 3 shows the selected models for each individual item (i.e., A-purging, B-loss-of-control eating, C-weight loss, D-feeling overweight, and E-attributing importance to food). All five models showed the best fit with two trajectory groups (i.e., high symptom levels and low symptom levels). The mean posterior probabilities were between 0.79 and 1.00, indicating a good fit. It can be noted that the trajectory patterns are similar for all items, particularly for the loss-of-control eating, feeling overweight and attributing importance to food symptoms. Indeed, the loss-of-control eating, weight loss, feeling overweight and attributing importance to food models (Fig. 3B, C, D, E) with the best fits had significant constant (low-level) and quadratic (high-level) trajectory groups (*P* < 0.001). The purging model (Fig. 3A) had two linear trajectory groups (low-level, *P* > 0.05; high-level, *P* < 0.05). Additionally, for the purging item, symptom levels were lower than for the other items (Fig. 3A**)**. For the weight loss item (Fig. 3C), a significant decrease in symptom level can be observed from ages 17 to 20 in the high-level trajectory group. Finally, the five low-level trajectory groups remained low and stable.

## Discussion

The present study investigated heterogeneity in ED symptoms from early adolescence to young adulthood in a large, longitudinal community sample, considering both sex and symptom type. Individuals in the trajectory group associated with high levels of symptoms consisted of a substantial part of the cohort (30.9%) and were already at higher risk of EDs at 12 years of age than those in the trajectory group associated with low levels of ED symptoms. Unsurprisingly, there were more girls in the trajectory group associated with high levels of ED symptoms than there were boys; however, boys and girls presented similar patterns of ED symptom trajectories. Trajectories by symptom types showed that some symptoms (i.e., purging) peak later in adolescence, while other symptoms (i.e., loss-of-control eating, feeling overweight, attributing importance to food) follow similar patterns and stay stable from 15  to 20 years.

Our findings support previous research that have found patterns with two or three trajectory groups of ED symptoms; typically a low-level trajectory group that remains low throughout adolescence, and a high-level trajectory group whose risk increases throughout adolescence [20, 22]. A new finding of the present study was that the most prominent rise in ED symptoms throughout adolescence occurred between 12 and 15 years of age. The preceding finding highlights that ED preventive interventions might not target signs and symptoms early enough [23, 24]. Specifically, most preventive intervention programs start in mid-adolescence or adulthood [24]. Indeed, Ciao et al. reviewed the literature on ED preventive programs, and a closer look at the included studies suggests that only one third of the programs targeted youth during middle school or early high school [24]. Our study highlights the importance of starting preventive interventions programs for EDs *prior to the onset of* adolescence, before ED symptoms start to increase. The rise in ED symptoms between ages 12 and 15 aligns with the beginning of puberty, which has been associated with physical, hormonal, and social changes [34, 35]. Moreover, there may be an increase in environmental stressors such as peer pressure around 12 to 15 years, which has been shown to influence the development of psychopathology including EDs, as young adolescents begin high school [36]. More research is needed to clarify the associations between these factors and ED symptoms evolution.

Another new finding of the present study is that, although girls were 1.3 times more likely to be in the high-level trajectory group than boys, ED symptom *patterns* from early adolescence to young adulthood were similar for boys and girls. Although girls also presented with more ED symptoms, our findings suggest that both boys and girls can already be at risk for developing EDs as early as 12 years old.

To further describe ED symptomatology over time, trajectories of specific symptoms were modeled. The weight loss item was the only symptom showing a decrease towards 17 to 20 years old. The removal of this symptom from the overall ED symptomatology model seemed to stabilize the trajectories and accounted for the decrease in ED symptoms at the end of adolescence. The weight loss item can be seen as a “physical” symptom, while other items of the SCOFF are more cognitive and behavioral, which may explain their distinctions. On this subject, Slane and colleagues [21] found that an increase in BMI was associated with an increase in cognitive symptoms (i.e., weight preoccupations, body dissatisfaction) related to EDs from late childhood to early adulthood. They also suggested that women gain weight faster at the beginning of adulthood than during other developmental periods, possibly explaining high levels of ED symptoms related to weight concerns and other ED cognitions at this age [21]. This represents a hypothesis to explain the decreasing pattern observed in the high-level trajectory group of the weight loss symptom reported here.

Being in the high-level trajectory group for the purging item of the SCOFF was less common than for other ED symptoms. This finding, as well as the proportion of individuals in the high-purging trajectory group (16.4%), is in line with previous data suggesting prevalence between 1–15% for purging symptoms in community samples of young adults [37–39]. Previous studies also considered other purging methods (e.g., diuretic and laxative use; [37–40]), however the purging item of the SCOFF is specific to vomiting. The high-purging trajectory group increased more slowly and peaked later in adolescence than the high-level trajectory groups for other SCOFF items. A few hypotheses can explain this. For example, it is possible to speculate that this specific behavior might be socially learned over time and might depend more on the social environment during adolescence than other symptoms. Supporting this hypothesis, previous studies have highlighted the role of peers and social environment in the development of purging behaviors during adolescence [41]. Also, the important environmental changes happening at the end of adolescence can represent stressful events for at-risk adolescents and might contribute to the peak of purging behaviors observed later in adolescence [42]. Overall, our findings indicate that purging behaviors, although indicative of significant disordered eating, may not be as common as other ED symptoms in young adolescents. Thus, the prevalence of purging behaviors should be considered when screening to detect disordered eating behaviors in this age group.

The loss-of-control eating, feeling overweight, and attributing importance to food symptoms all had relatively similar trajectories, with high proportions of individuals presenting with high-level of those behaviors, and relatively stable trajectories from 15 to 20 years. Almost half of the sample was in the high-level trajectory group for the loss-of-control eating (40.9%) and the attributing importance to food (53.9%) symptoms. These three items (i.e., loss-of-control eating, feeling overweight, and attributing importance to food) might be somewhat related, possibly through their link with cognitions related to body image and weight concerns [43–45]. Both feeling overweight and attributing importance to food can be considered maintenance factors for binge-purging behaviors, while loss-of-control eating is a component of binge eating [4, 46, 47]. Thus, these factors could represent important preventive and therapeutic treatment targets in EDs and need to be further studied.

Strengths of the current study are the use of a relatively large, community-based sample that included boys and girls, which improves the generalizability of the findings, as well as the long period of data collection that covered all adolescence, starting at 12 years. A few limitations however need to be considered when interpreting the results. First, due to the research objective of the present study, which was to qualitatively describe the heterogeneity of ED symptom development in the QLSCD using a statistical basis, we opted for the Proc Traj procedure. The latter has been widely used over the years and is a practical choice given its relative ease of use and interpretation [30, 48–50]. However, we are aware that a limitation of this approach compared to others (e.g. growth mixture models, which also have their own drawbacks) is that it does not take into account inter-individual variability within a trajectory group (i.e. it is fixed at 0; [48]). A second limitation concerns the use of data from individuals in the community; therefore, they might not be generalizable to clinical populations. Third, as is often the case with longitudinal studies, there was a substantial amount of missing data at the different timepoints in adolescence, particularly in boys. However, this was handled using maximum likelihood estimation. Fourth, cultural differences might exist in ED symptom presentations and this was not assessed in the current study. Future studies should investigate developmental trajectories of ED symptoms in a more ethnically diverse sample as done in the present study. Fifth, the SCOFF is a self-reported questionnaire, and some items might be open to interpretation during assessment, which could have induced a bias. The SCOFF does not assess all types of disordered eating behaviors, with some possibly being more common in the general population (e.g., excessive exercise, dietary restraint, emotional eating). Lastly, the dichotomization of the SCOFF score as recommended by previous studies [28, 29] might have led to a loss of information in the trajectory model on overall levels of eating disorder symptoms. On the other hand, the estimation of separate trajectories for the five eating disorder symptoms assessed by the SCOFF allowed us to qualitatively examine the evolution of each SCOFF symptom across time in the QLSCD cohort.

## Conclusions

This study identified different trajectory groups based on individual’s ED symptom levels from early adolescence to young adulthood, in a community cohort. Our findings indicate a peak in ED symptom levels between 12 and 15 years of age—a period where most ED prevention programs have not started yet. Although ED prevention programs during high school have been shown to be effective in adolescents, results of our study suggest that these programs may not be offered early enough to prevent EDs in the most efficient way, considering at-risk individuals already have elevated ED symptoms at 12 years of age. Thus, our findings highlight the need for the development of ED prevention programs specifically designed for children, before they enter adolescence and develop the first signs of EDs. Such early interventions, in turn, will help to prevent full-blown EDs and their associated negative outcomes.

## Supplementary Information

**Additional file 1.** Model fit statistics and group membership for each retained trajectory model.

**Additional file 2.** Eating disorder symptom level trajectories from 12 to 20 years, including both sex (without the weight loss item).

## Data Availability

The data that support the findings of this study are available from The Research Unit on Children’s Psychosocial maladjustment (GRIP) but restrictions apply to the availability of these data, which were used under license for the current study, and so are not publicly available. Data are however available from the authors upon reasonable request and with permission of the GRIP.

## Abbreviations

ED: Eating disorders
AN: Anorexia nervosa
BN: Bulimia nervosa
BED: Binge eating disorder
BMI: Body mass index
QLSCD: Quebec Longitudinal Study of Child Development
SCOFF: Sick, Control, One Stone, Fat, Food
BIC: Bayesian Information Criterion
