# Supplementary material for: Developmental trajectories of eating disorder symptoms: A longitudinal study from early adolescence to young adulthood
Source: J Eat Disord. 2022 Jun 20;10:84. doi: 10.1186/s40337-022-00603-z (PMC9210773; doi:10.1186/s40337-022-00603-z)
Supplement: Supplementary file 1 — Additional file 1. Model fit statistics and group membership for each retained trajectory model. [file 40337_2022_603_MOESM1_ESM.pptx]

## Slide 1
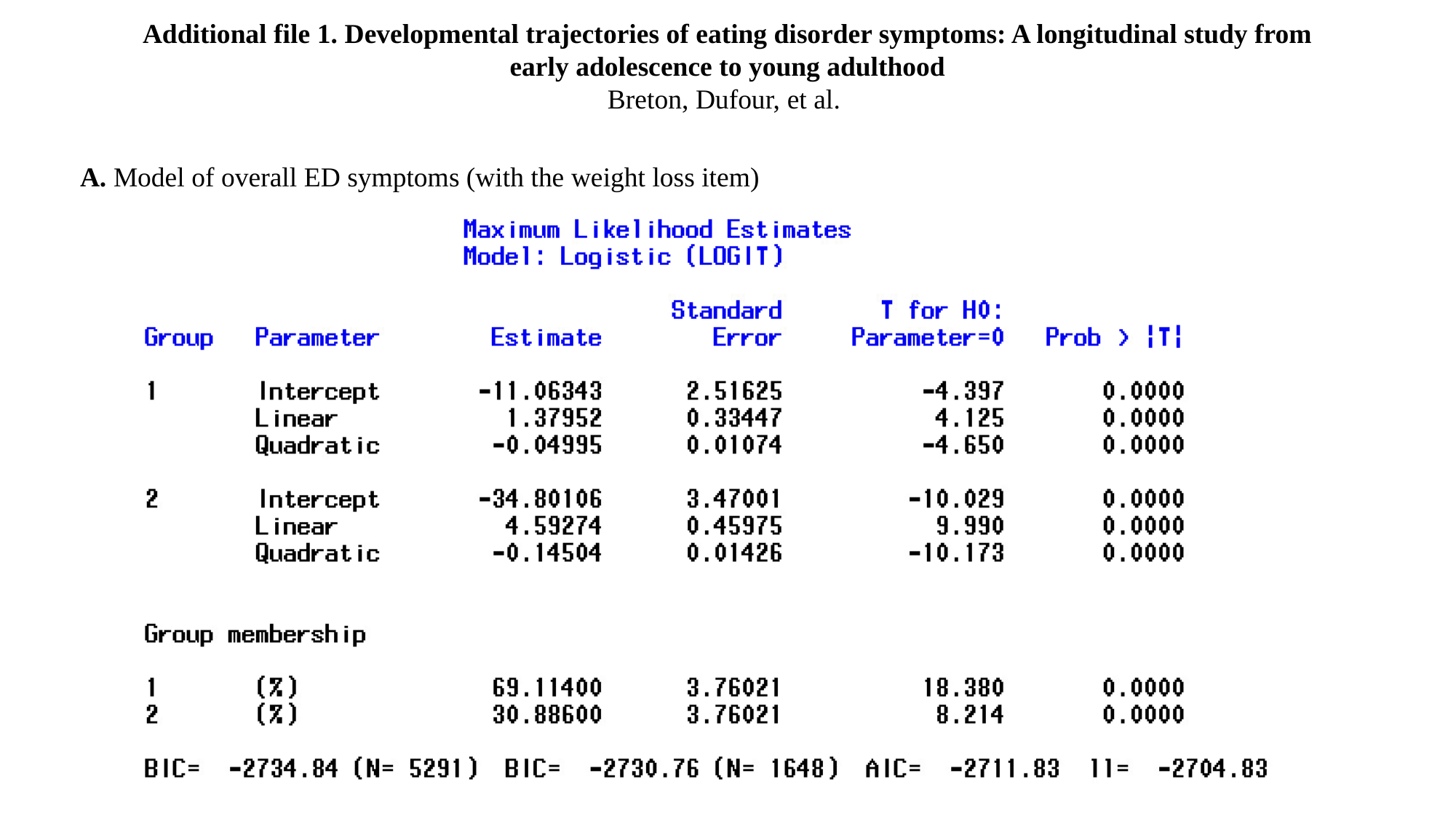

Additional file 1. Developmental trajectories of eating disorder symptoms: A longitudinal study from early adolescence to young adulthood
Breton, Dufour, et al.
A. Model of overall ED symptoms (with the weight loss item)

## Slide 2
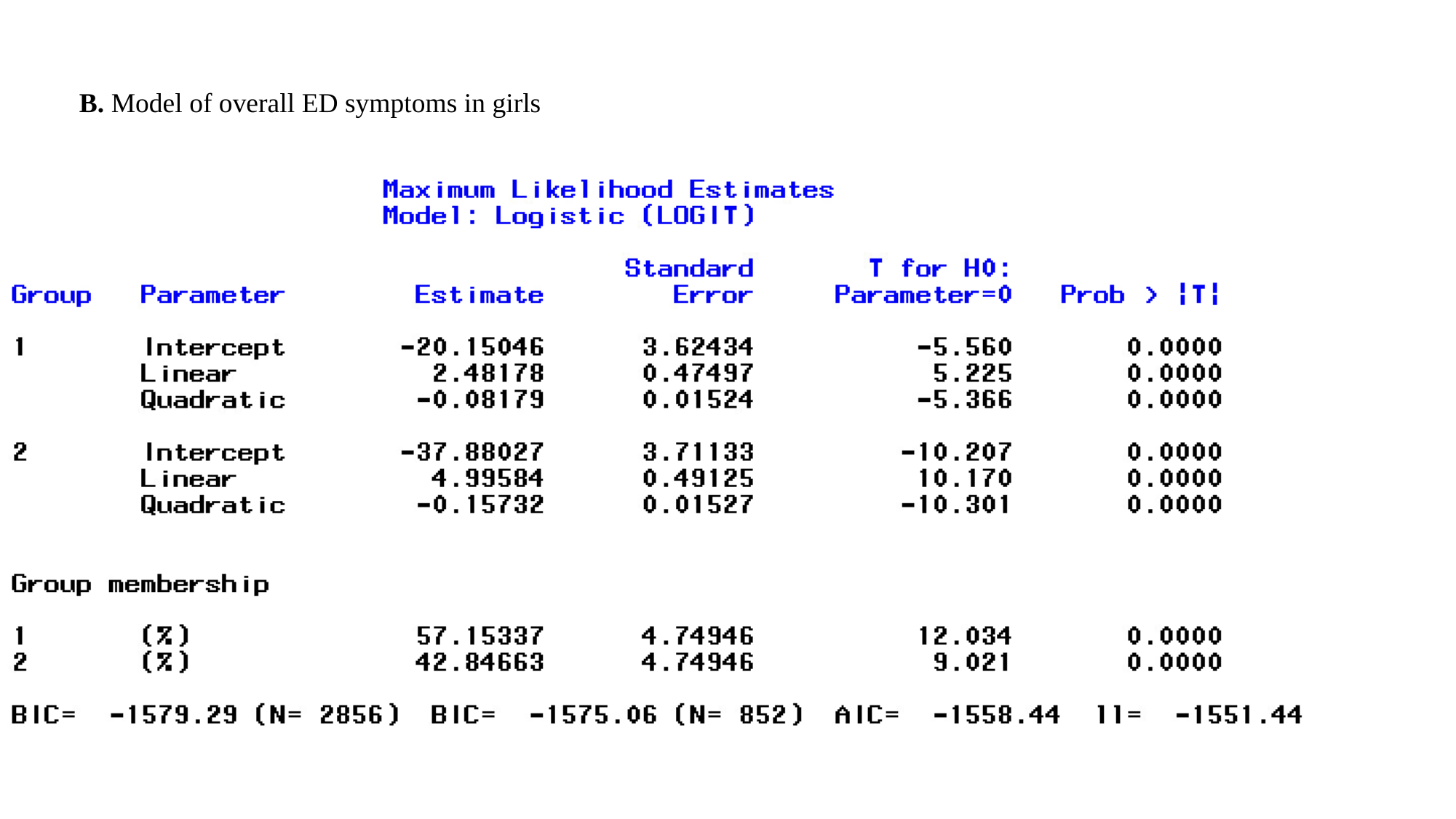

B. Model of overall ED symptoms in girls

## Slide 3
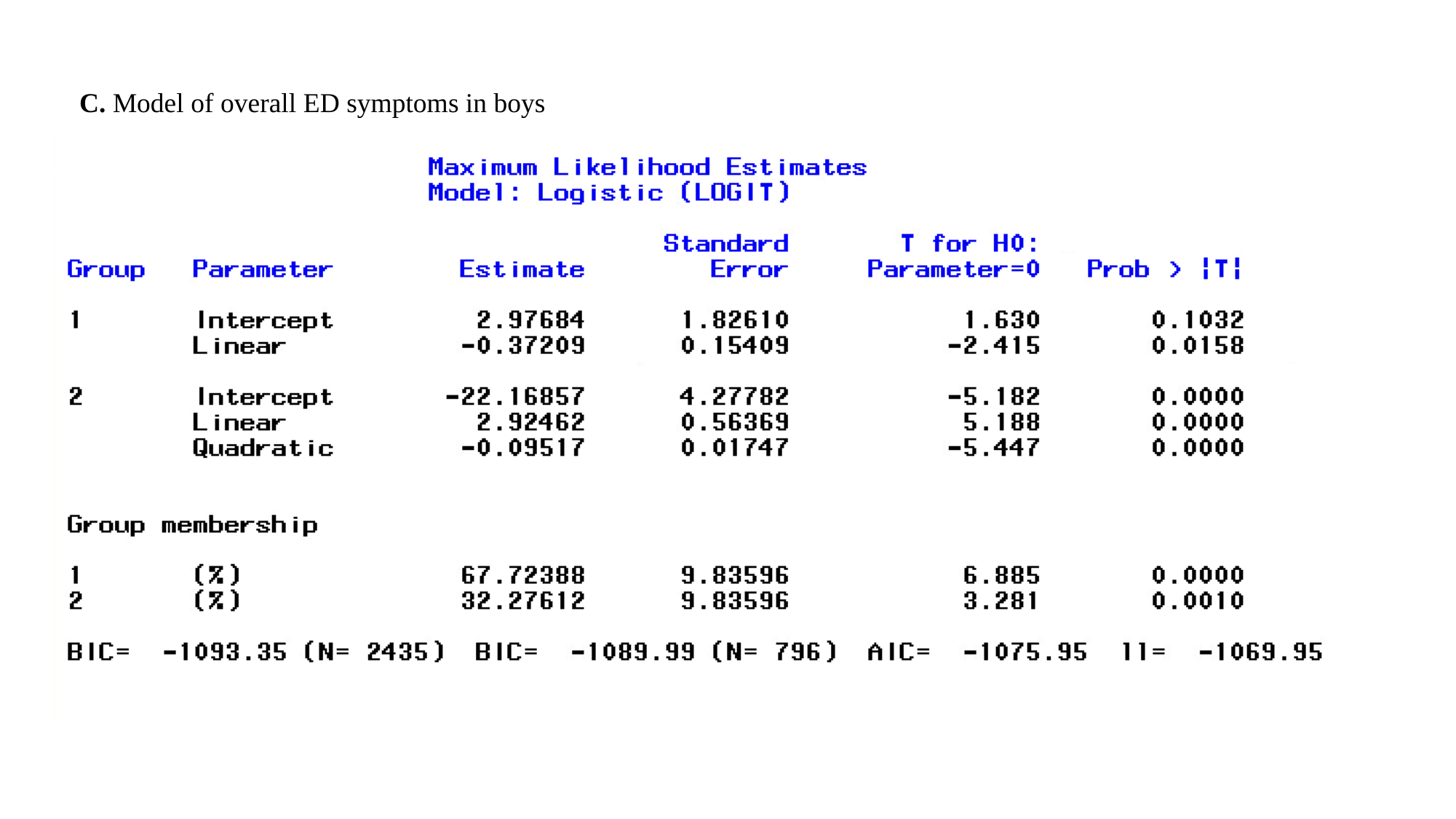

C. Model of overall ED symptoms in boys

## Slide 4
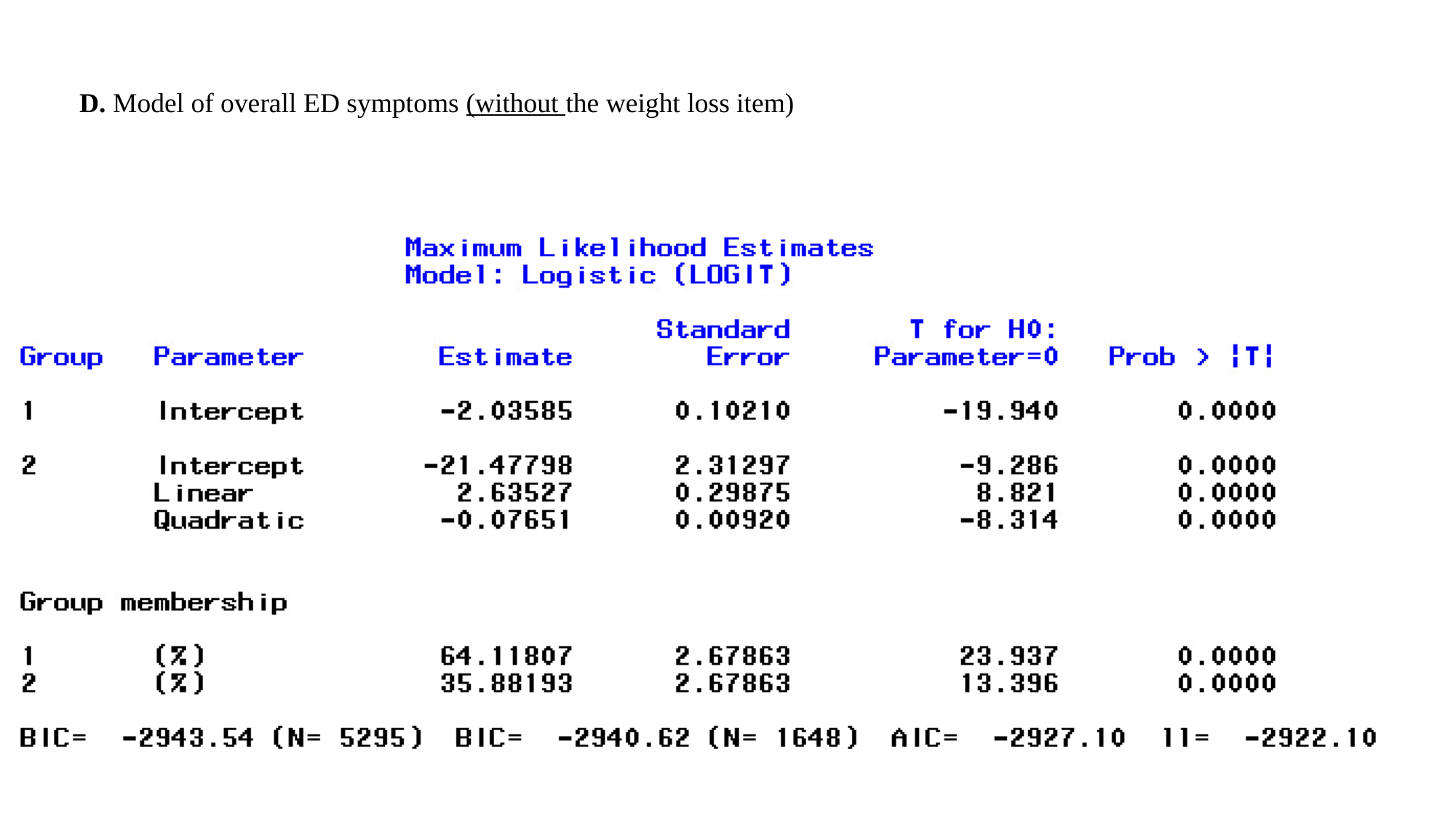

D. Model of overall ED symptoms (without the weight loss item)

## Slide 5
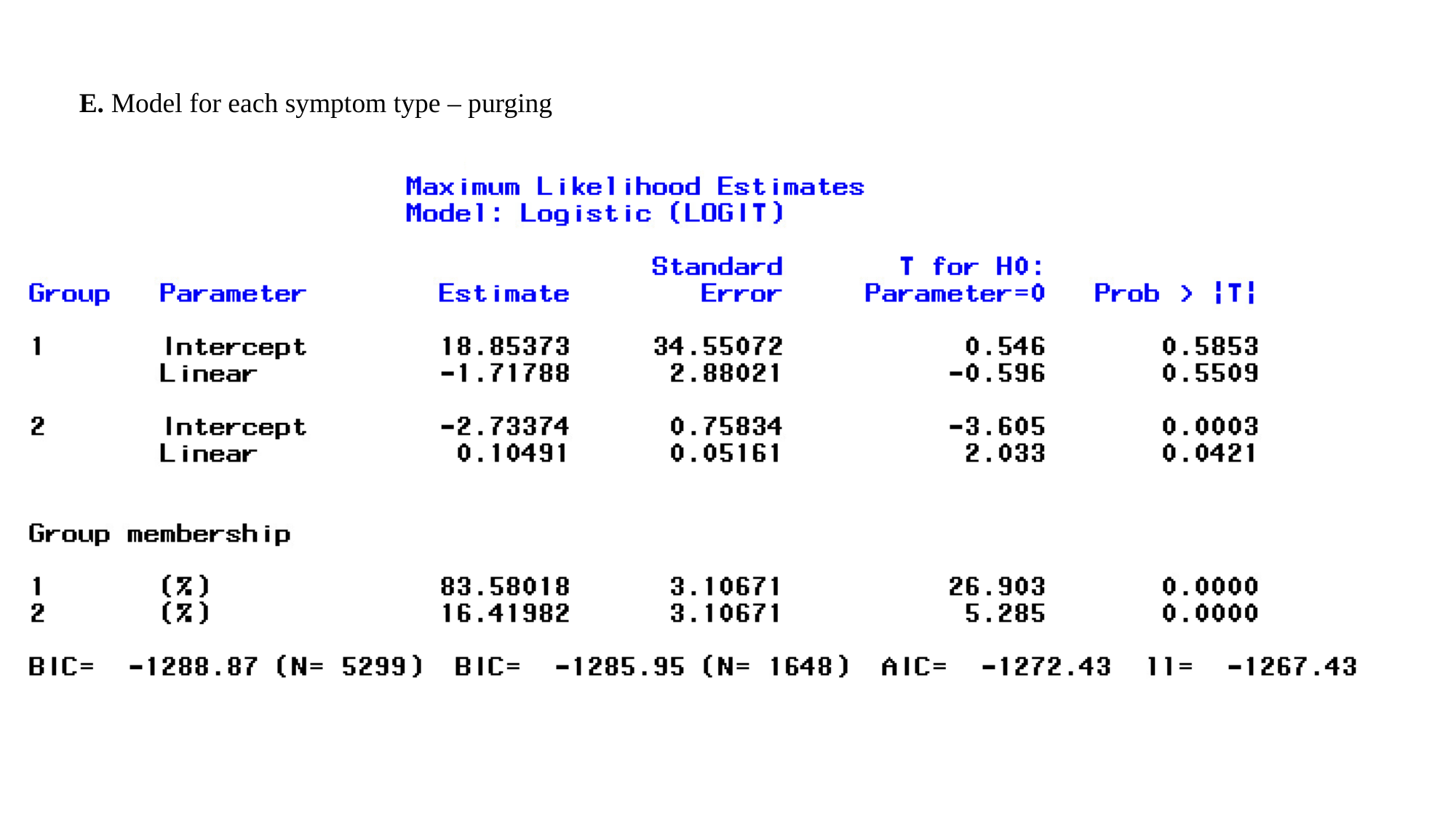

E. Model for each symptom type – purging

## Slide 6
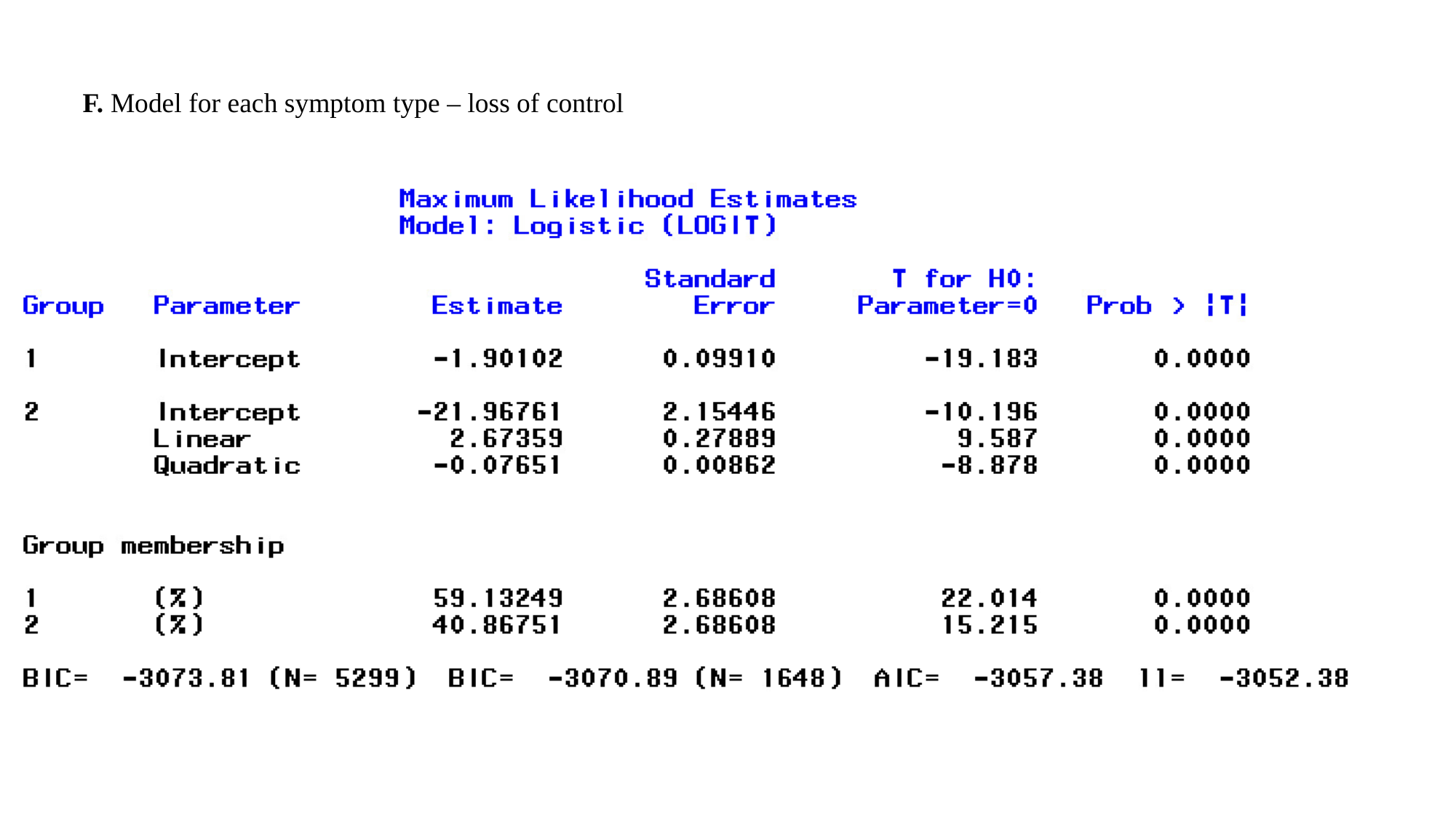

F. Model for each symptom type – loss of control

## Slide 7
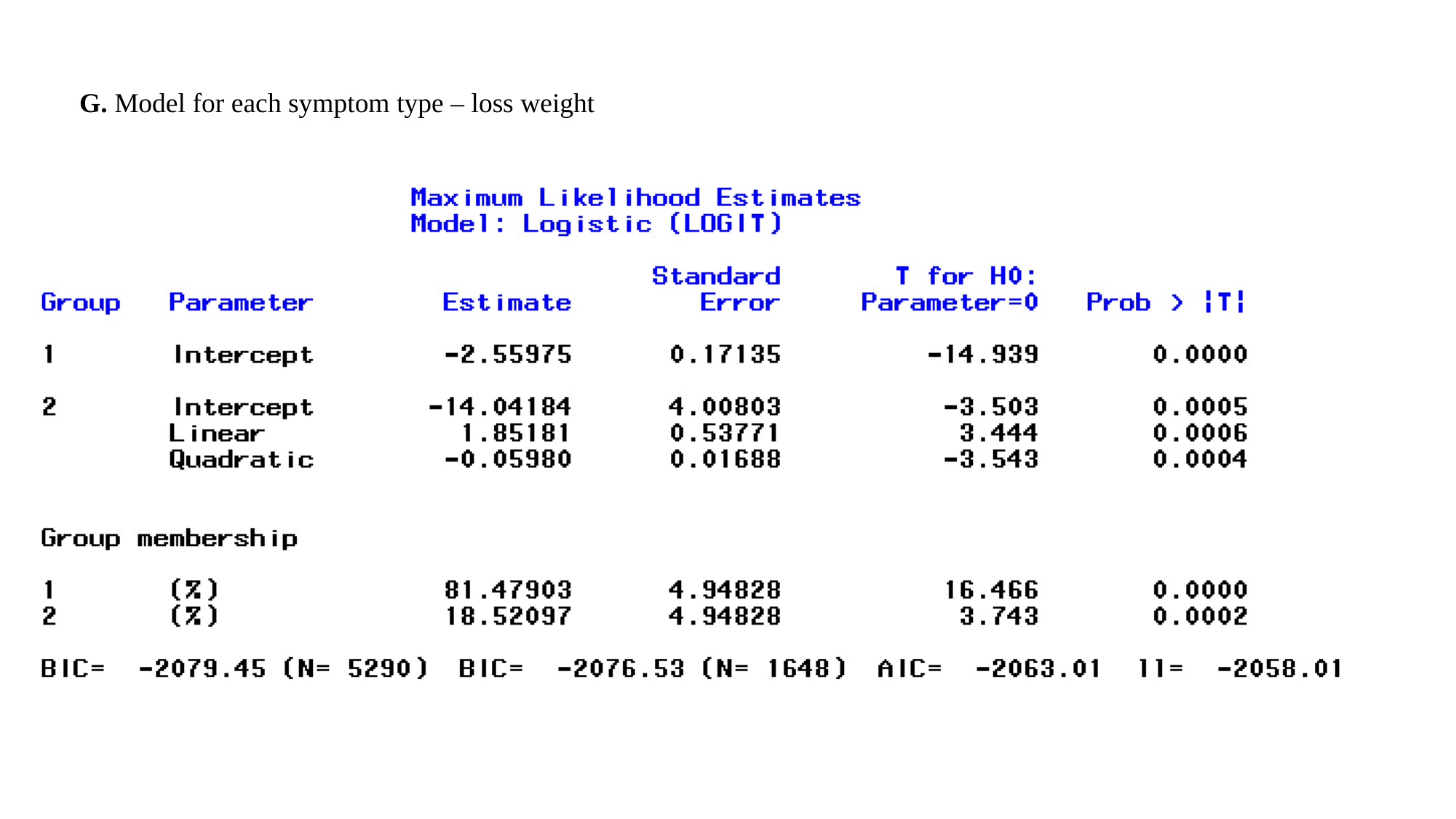

G. Model for each symptom type – loss weight

## Slide 8
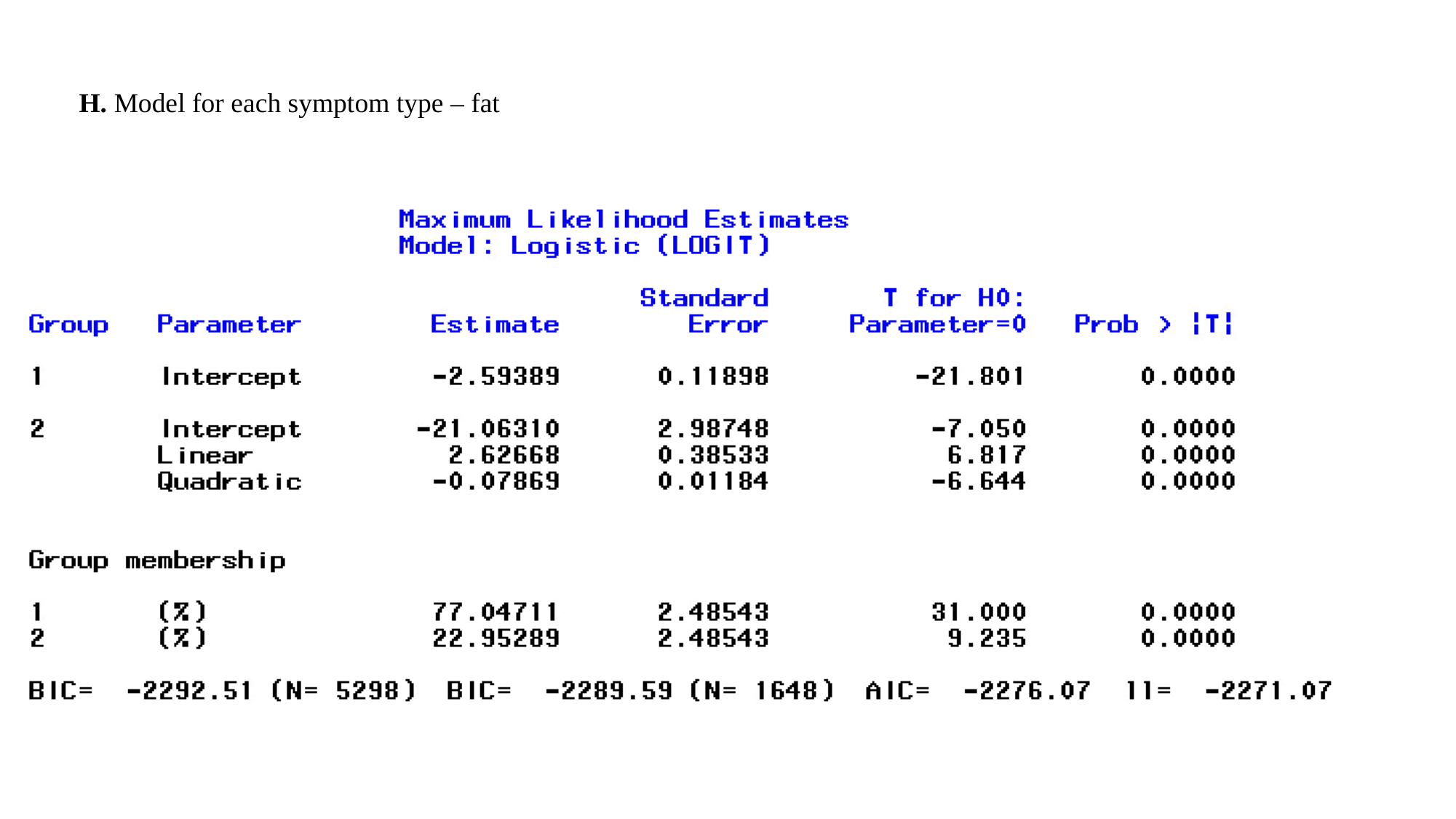

H. Model for each symptom type – fat

## Slide 9
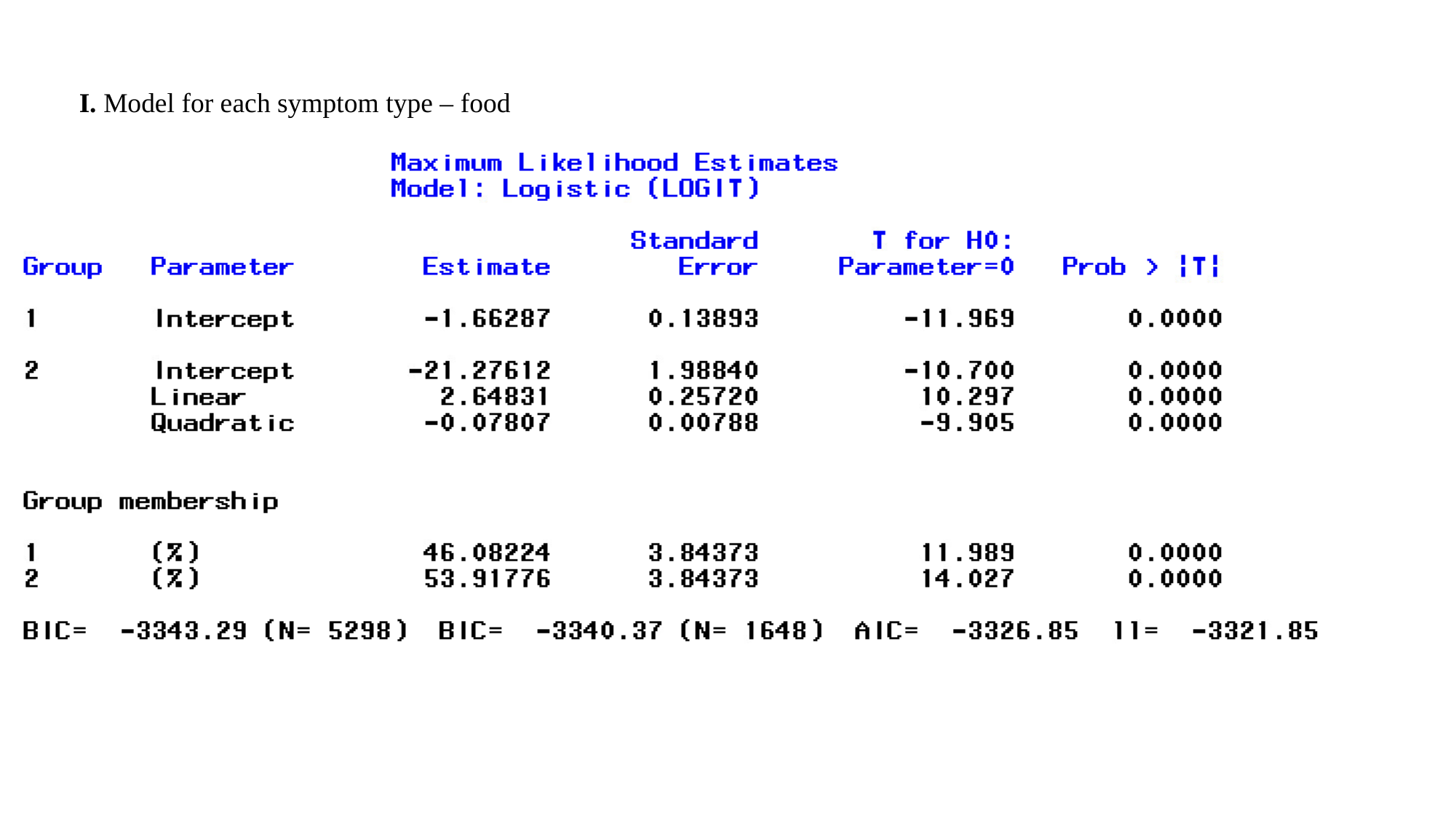

I. Model for each symptom type – food
