# Supplementary material for: Developmental trajectories of eating disorder symptoms: A longitudinal study from early adolescence to young adulthood
Source: J Eat Disord. 2022 Jun 20;10:84. doi: 10.1186/s40337-022-00603-z (PMC9210773; doi:10.1186/s40337-022-00603-z)

**Additional file 2.** **Developmental trajectories of eating disorder symptoms: A longitudinal study from early adolescence to young adulthood**

Breton, Dufour, et al.


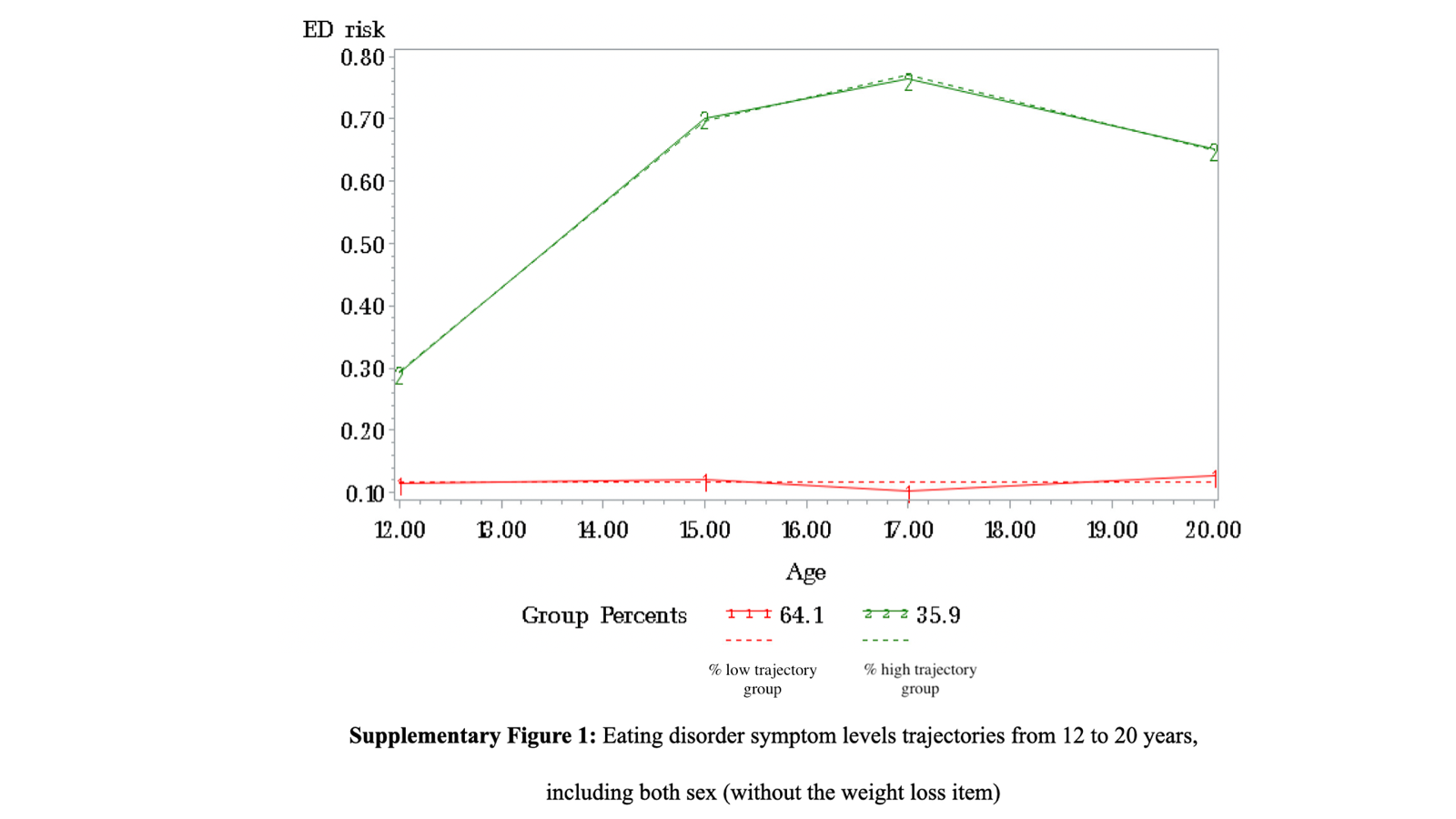

Supplement: Supplementary file 2 — Additional file 2. Eating disorder symptom level trajectories from 12 to 20 years, including both sex (without the weight loss item). [file 40337_2022_603_MOESM2_ESM.docx]
